# COVID-19 Transmission Within a Family Cluster in Yancheng, China

**DOI:** 10.3389/fmed.2020.00387

**Published:** 2020-07-03

**Authors:** Hongming Zhang, Runzhe Chen, Jibei Chen, Baoan Chen

**Affiliations:** ^1^Department of Respiratory Medicine, Yancheng Third People's Hospital, The Affiliated Yancheng Hospital of Southeast University Medical College, Yancheng, China; ^2^Department of Hematology and Oncology, Medical School, Zhongda Hospital, Southeast University, Nanjing, China; ^3^Department of Thoracic/Head & Neck Medical Oncology, University of Texas MD Anderson Cancer Center, Houston, TX, United States

**Keywords:** COVID-19, SARS-CoV-2, family cluster, asymptomatic, transmission, intimate contact

## Abstract

We report the clinical features of novel coronavirus disease 2019 (COVID-19) caused by severe acute respiratory syndrome coronavirus 2 (SARS-CoV-2) infection in a family setting of 13 people with person-to-person transmission in Yancheng, Jiangsu Province, China.

## Introduction

Since the first case of a novel coronavirus disease 2019 (COVID-19) was detected in early December 2019, it has spread rapidly all over the world (1). As of June 17, 2020, more than 8 million of confirmed cases with 440,000 deaths have been reported globally (2). Several family clusters of infected individuals have been reported, which presents a serious threat to public health (3–6). In previously reported family clusters, most infected individuals have exhibited clinical symptoms, abnormal lymphocyte counts, and chest computed tomography (CT) images and were positive for the severe acute respiratory syndrome coronavirus 2 (SARS-CoV-2) on quantitative RT-PCR (qRT-PCR) assays. However, some patients were found to have lung abnormalities on chest CT images and positive qRT-PCR results without any clinical symptoms. Here, we report the clinical characteristics of COVID-19 in a family setting of 13 people with person-to-person transmission in Yancheng, Jiangsu Province, China.

## Methods

Data were collected from Yancheng Third People's Hospital of Jiangsu Province, China. A total of 13 patients from a family cluster were tested SARS-CoV-2 positive after seven of the family members had been to Wuhan. Patients were hospitalized from January 26, 2020 to February 28, 2020. Throat swab samples were collected, and SARS-CoV-2 was detected using qRT-PCR assay. CT and hematological examinations were performed. Patients were carefully monitored and treated during hospital isolation. This study was approved by the ethics committee of Yancheng Third People's Hospital of Jiangsu Province, and written informed consent was obtained. This study followed the reporting guideline for case series.

## Results

**Figure 1:**
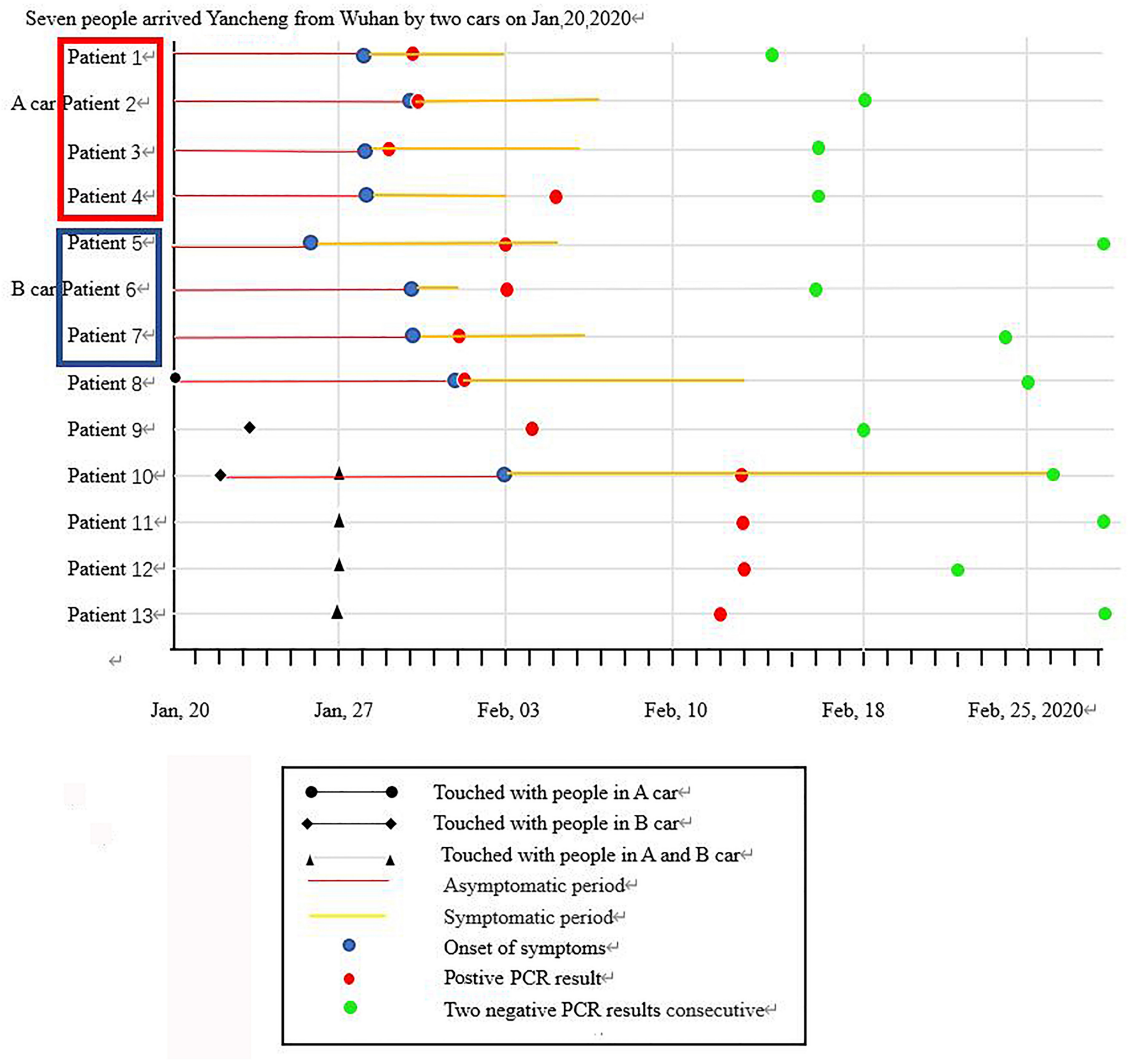
Timeline of SARS-CoV-2 infection in a familial cluster.

**Figure 2:**
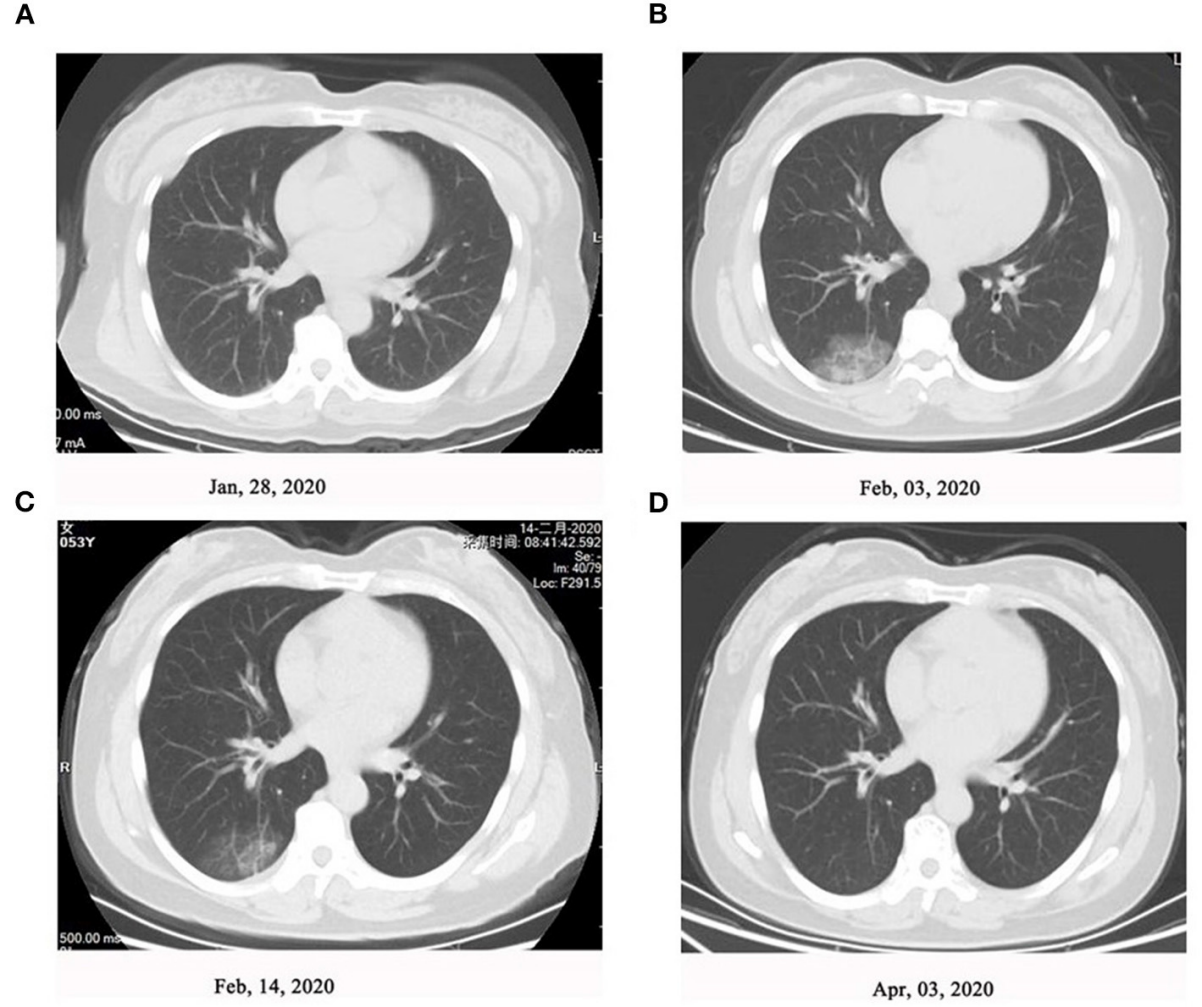
Representative changes of chest computed tomography (CT) images in Patient 3. High-resolution chest CT scan showed **(A)** normal chest at level of left atrium (January 28, 2020), **(B)** ground-glass attenuation close to the pleura in the right lower lung (February 03, 2020), **(C)** fade of ground-glass attenuation after treatment (February 14, 2020), and **(D)** resolution of lung inflammation after treatment (April 03, 2020).

As shown in Figure 1, on January 20, 2020, seven family members without any symptoms went back to Yancheng from Wuhan via two cars after participating in activities celebrating the Chinese Spring Festival. In Yancheng, two people from this family were infected after touching one or more out of the seven people at home on January 20 and January 23, respectively. Another four family members were subsequently infected following a family wedding together with the above nine people on January 27. Afterwards, all the 13 family members were tested positive for SARS-CoV-2 infection. Of these 13 patients, six cases had fever, two cases had cough, and one had fatigue and dizziness as the first manifestations; however, case did not present with any symptoms (Table 1). Nine patients developed symptoms after an average of 9 days of exposure, and those four asymptomatic patients tested as qRT-PCR positive after exposure for an average of 15.5 days. CT scans of 10 patients when admitted showed mild or moderate pulmonary fibrosis, but no abnormalities were observed in three patients by chest CT images. During the hospital isolation ward stay, all patients were carefully monitored. Besides supportive oxygen therapy, all the adult patients were treated with intramuscular thymalfasin (1.6 mg per day to twice per week according to patients' response), oral hydroxychloroquine (0.4 mg per day for 7 days), alpha interferon (nebulized inhalation, 500 iu twice per day for 10 days), and oral lopinavir/ritonavir (500 mg twice per day for 6–16 days). All patients were also received traditional Chinese medicine including oral Lianhua Qingwen Granules (1.4 g three times/day for 6–13 days), oral Shufeng Jiedu Capsules (2 g three times/day for 6–13 days), and oral Arbidol (0.2 g three time/day for 6–13 days). No obvious adverse effects of these agents were observed; an exception was an 88-year-old with mild dizziness and unstable walking after taking hydroxychloroquine. All these 13 patients in the family recovered well with symptom-free and pulmonary fibrosis was absorbed by chest CT and qRT-PCR re-evaluations until discharge after around 2 months of hospitalization. Representative changes of chest CT images in Patient 3 were presented in Figure 2 during this time course.

**Table 1:** Clinical characteristics of 13 COVID-19 infected patients.

**Serial number**	**Gender**	**Age**	**Comorbidity**	**Epidemiological history**	**Clinical manifestations**
Patient 1	Male	30	None	Arrived Yancheng from Wuhan by A car on Jan 20, 2020	Dizziness, fatigue, fever
Patient 2	Male	53	None	Arrived Yancheng from Wuhan by A car on Jan 20, 2020	Fever, itchy throat
Patient 3	Female	54	None	Arrived Yancheng from Wuhan by A car on Jan 20, 2020	Fever, fatigue
Patient 4	Male	7	None	Arrived Yancheng from Wuhan by A car on Jan 20, 2020	Dizziness, fatigue, fever
Patient 5	Male	70	None	Arrived Yancheng from Wuhan by B car on Jan 20, 2020	Fever
Patient 6	Female	46	None	Arrived Yancheng from Wuhan by B car on Jan 20, 2020	Dry cough
Patient 7	Male	22	None	Arrived Yancheng from Wuhan by B car on Jan 20, 2020	Itchy throat, fever
Patient 8	Female	77	None	Touched with people in A car from Jan 23. Attended a wedding on Jan 27–28, 2020	Fever
Patient 9	Male	48	None	Touched with patient 5 from Jan 23, 2020. Attended a wedding on Jan 27–28, 2020	No symptoms
Patient 10	Male	47	None	Touched with patient 5 from Jan 22, 2020. Attended a wedding on Jan 27–28, 2020	Cough
Patient 11	Male	87	Hypertension, chronic bronchitis, pulmonary emphysema	Attended a wedding on Jan 27–28, 2020	No symptoms
Patient 12	Male	17	None	Attended a wedding on Jan 27–28, 2020	No symptoms
Patient 13	Male	63	Hypertension diabetes, mellitus	Attended a wedding on Jan 27–28, 2020	No symptoms

## Discussion

We reported a cluster of 13 family members of infected with SARS-CoV-2. The uniqueness of this cluster is that only four people were infected during the wedding with so many people attending the wedding. Therefore, it has been assumed that infection of this virus is correlated with the strength of individual immunity (7, 8). Furthermore, although all individuals were tested positive for SARS-CoV-2 infection on qRT-PCR in this family cluster, four patients did not show any clinical symptoms and diagnosis may have been delayed owing to atypical presentations. Asymptomatic patients might be unaware of their disease and therefore not isolate themselves or seek further treatment, or they might be overlooked by health-care professionals and thus unknowingly transmit the virus to others (3, 8, 9). Fortunately, symptoms of the four asymptomatic patients in this family cluster were mild, and all of them were recovered. However, to prevent and control this highly infectious disease as early as possible, people with family members with COVID-19 diagnosis should be closely monitored and tested to rule out the virus infection, even if they do not show any symptoms. It is also important for countries to do active case-findings among close contacts of confirmed patients to prevent symptoms worsen and virus spreading (10). Finally, Chinese medicine was used to as part of the treatment against COVID-19 in this family cluster with good self-reported feedback and no obvious adverse effects, thus recommending a consideration of its use according to our experience, though concrete mechanisms still need further investigation (11).

## Data Availability Statement

The raw data supporting the conclusions of this article will be made available by the authors, without undue reservation.

## Ethics Statement

The studies involving human participants were reviewed and approved by Yancheng Third People's Hospital. Written informed consent to participate in this study was provided by the participants' legal guardian/next of kin. Written informed consent was obtained from the individual(s), and minor(s)' legal guardian/next of kin, for the publication of any potentially identifiable images or data included in this article.

## Author Contributions

HZ, JC, RC, and BC: data collection and interpretation. RC and HZ: original draft preparation. RC and BC: review and editing. BC, RC, and JC: supervision. All authors: reviewed and approved the final version of the manuscript.

## Conflict of Interest

The authors declare that the research was conducted in the absence of any commercial or financial relationships that could be construed as a potential conflict of interest.
